# Cerebral Blood Flow in Community-Based Older Twins Is Moderately Heritable: An Arterial Spin Labeling Perfusion Imaging Study

**DOI:** 10.3389/fnagi.2019.00169

**Published:** 2019-07-02

**Authors:** Jiyang Jiang, Anbupalam Thalamuthu, Forrest C. Koch, Tao Liu, Qun Xu, Julian N. Trollor, David Ames, Margaret J. Wright, Vibeke Catts, Perminder S. Sachdev, Wei Wen

**Affiliations:** ^1^Centre for Healthy Brain Ageing, School of Psychiatry, University of New South Wales, Randwick, NSW, Australia; ^2^Neuroscience Research Australia, Randwick, NSW, Australia; ^3^School of Biological Science and Medical Engineering, Beihang University, Beijing, China; ^4^Department of Health Manage Centre, Renji Hospital, School of Medicine, Shanghai Jiao Tong University, Shanghai, China; ^5^Renji-UNSW CHeBA Neurocognitive Centre, Renji Hospital, School of Medicine, Shanghai Jiao Tong University, Shanghai, China; ^6^Neuropsychiatric Institute, Prince of Wales Hospital, Randwick, NSW, Australia; ^7^Department of Developmental Disability Neuropsychiatry (3DN), University of New South Wales, Randwick, NSW, Australia; ^8^National Ageing Research Institute, University of Melbourne, Parkville, VIC, Australia; ^9^NeuroImaging Genetics Laboratory, QIMR Berghofer Medical Research Institute, Herston, QLD, Australia; ^10^Queensland Brain Institute, The University of Queensland, Brisbane, QLD, Australia

**Keywords:** cerebral blood flow, heritability, twin study, community-dwelling, aging

## Abstract

Adequate cerebral blood flow (CBF) is necessary to maintain brain metabolism and function. Arterial spin labeling (ASL) is an emerging MRI technique offering a non-invasive and reliable quantification of CBF. The genetic basis of CBF has not been well documented, and one approach to investigate this is to examine its heritability. The current study aimed to examine the heritability of CBF using ASL data from a cohort of community-dwelling older twins (41 monozygotic (MZ) and 25 dizygotic (DZ) twin pairs; age range, 65–93 years; 56.4% female). The results showed that the cortex had higher CBF than subcortical gray matter (GM) regions, and CBF in the GM regions of the anterior cerebral artery (ACA) territory was lower than that of the middle (MCA) and posterior (PCA) cerebral arteries. After accounting for the effects of age, sex and scanner, moderate heritability was identified for global CBF (*h*^2^ = 0.611; 95% CI = 0.380–0.761), as well as for cortical and subcortical GM and the GM in the major arterial territories (*h*^2^ = 0.500–0.612). Strong genetic correlations (GCs) were found between CBF in subcortical and cortical GM regions, as well as among the three arterial territories (ACA, MCA, PCA), suggesting a largely convergent genetic control for the CBF in brain GM. The moderate heritability of CBF warrants future investigations to uncover the genetic variants and genes that regulate CBF.

## Introduction

Accounting for ~20% of body’s oxygen consumption for normal function (Attwell and Laughlin, 2001), the brain is the most metabolically active human organ. Due to the limited intracellular energy storage within neurons, adequate cerebral blood flow (CBF) is essential to maintain neuronal metabolism and brain function. Available findings on the age effects on CBF have been inconsistent. Across the lifespan, some studies have suggested that CBF tends to stabilize after a significant drop at approximately 16 years of age (Biagi et al., 2007), while others showed a constantly decreasing CBF with age from 20 s to 80 s (Lu et al., 2011). Other studies investigating the differences in CBF between young and elderly adults have also shown mixed findings (Dastur, 1985; Xu et al., 2010). Most studies focusing on normal aging participants have reported an age-related decrease in CBF (Zhang et al., 2017; Tarumi and Zhang, 2018), although the age effects on cerebral metabolic rate of oxygen (i.e., oxygen demand for metabolism) are inconsistent (Marchal et al., 1992; Lu et al., 2011). Heterogeneity in aging-induced CBF alteration has been observed across brain regions, but the spatial pattern varies among studies (Lee et al., 2009; Chen et al., 2011). The reasons underlying the CBF reduction in aging are still largely unknown, but impaired cardiovascular function (Tarumi and Zhang, 2018) and disrupted CBF autoregulation (Cipolla, 2009) may contribute to the decline.

A few studies have shown that genetic factors are involved in regulating CBF. APOE ε4 genotype was shown to differentially affect CBF across the lifespan (Wierenga et al., 2013). A genetic variant for ATP-binding cassette sub-family C member 9 (ABCC9), rs704180, has been associated with age-related hippocampal sclerosis and cerebral arteriolosclerosis. Individuals with the risk alleles (AA) on rs704180 also showed an average of 28% decrease in global CBF when compared to those with non-risk alleles (GG, Ighodaro et al., 2017). However, our understanding on the genetic basis of CBF and its changes is still limited. Particularly, the extent to which genetic factors control the CBF in aging has not been well documented. So far only two studies investigated the heritability of CBF. One study using flow alternating inversion recovery (FAIR) data showed a CBF heritability of 0.31 for cerebral gray matter (GM) in a cohort of siblings aged 40–90 years (Raffield et al., 2015). The other study, using phase-contrast MRI angiographic scout imaging, examined CBF rate (mL/s) and velocity (cm/s), as well as cross-sectional vessel area, within manually labeled basilar and bilateral carotid arteries in a sub-sample of the Rotterdam Study [*N* = 4,472; age = 64.8 ± 10.8 years; (Ikram et al., 2018)]. Using genotyping data, the study showed that CBF rate in basilar artery had the highest heritability (*h*^2^ = 0.218), followed by total CBF rate (*h*^2^ = 0.161). It is noteworthy that although both these two studies investigated CBF, the measures represented two different aspects of CBF. FAIR is a symmetrical arterial spin labeling (ASL) technique measuring the perfusion of blood into brain tissue, whereas MR angiography measures CBF velocity within vessels. So far, there has not been any studies investigating the heritability of CBF using twin samples. Twin studies allow for more accurate modeling of genetic and environmental factors, and are often considered as the optimal approach in estimating the genetic basis of a phenotype.

The two major arterial blood supply routes, the internal carotid arteries and the vertebral arteries, serve the anterior and posterior cerebral circulation, respectively (Farkas and Luiten, 2001). Anterior (ACA) and middle (MCA) cerebral arteries emanate from the internal carotid arteries. ACA is responsible for the midline portion of frontal lobe and superior medial parietal region, as well as anterior part of the limbic system. MCA supply oxygenated blood to the rest of frontal and parietal regions that ACA does not cover, superior and medial parts of the temporal lobe, and basal ganglia. Posterior cerebral artery (PCA), extended from vertebral arteries, irrigates the occipital lobe, the inferior part of temporal lobe, and thalami. In addition, some cerebrovascular diseases may favor a particular artery. For example, the underlying cause of an ischaemic stroke is often blocked carotid arteries (Coutinho et al., 2017). As a result, it would be interesting to investigate the differences in genetic controls between the three arterial territories. Moreover, deep GM regions, such as basal ganglia, have dedicated arterial branches to supply oxygenated blood, including lenticulostriate and anterior choroidal arteries, as well as penetrating branches from PCA. This has led us to study the differences in genetic influences between the CBF of cortical and subcortical GM regions.

ASL is a perfusion MRI sequence enabling quantification of CBF (Alsop et al., 2015). It offers a reliable and non-invasive alternative to H_2_^15^O positron emission tomography (PET), the gold standard for CBF quantification (Xu et al., 2010). Magnetically labeled blood water protons are used as endogenous tracer for CBF measurements. The FAIR imaging mentioned above is an easy-to-implement and relatively straightforward ASL sequence first introduced in mid-1990s. FAIR suffers from artifacts due to the imperfections in the profile of the inversion slice which causes problematic multi-slice applications (Borogovac and Asllani, 2012). Pseudo-continuous (PCASL) and (asymmetrical) pulsed (PASL) ASL are two proton labeling techniques that are currently widely implemented in ASL perfusion imaging. While PCASL applies thousands of shaped radiofrequency pulses to a single labeling plane, PASL employs one or a limited number of pulses to label a thick labeling slab (Alsop et al., 2015). PCASL is considered to have higher signal-to-noise ratio (SNR) compared to PASL due to the longer labeling duration, higher labeled magnetization, and less T1 decay (Alsop et al., 2015).

In the current study, we aimed to identify the heritability of CBF in a cohort of older twins using the latest ASL perfusion imaging techniques (both PCASL and PASL). We also examined the heritability and genetic correlations (GCs) of regional CBF in the three major arterial territories, as well as in the cortex and subcortical GM regions.

## Materials and Methods

### Study Sample

The study sample was drawn from the Older Australian Twins Study (OATS; Sachdev et al., 2009), a longitudinal study of twins aged 65 years and above living in three states of Australia, namely New South Wales, Victoria and Queensland. The participants were primarily recruited from the Australian Twin Registry. The zygosity of each twin pair was confirmed by genotyping on high-density single nucleotide polymorphism arrays. OATS has been approved by the ethics committees of the Australian Twin Registry, University of New South Wales, University of Melbourne, Queensland Institute of Medical Research, and the South Eastern Sydney & Illawarra Area Health Service. Informed consent was obtained from all participants, and the methods were carried out in accordance with the relevant guidelines. Forty-one monozygotic (MZ) and 25 dizygotic (DZ) twin pairs with ASL perfusion imaging and T1-weighted scans were included in the current study (Table 1 and Supplementary Table S1).

**Table 1:** Sample characteristics.

	MZ	DZ	*F* or chi-square	*p*-value
*N*	82 (41 pairs)	50 (25 pairs)	-	-
Age (years)	72.75 ± 5.08 (range, 65.37–88.30)	73.13 ± 4.68 (range, 65.69–93.31)	0.117	0.733
%Female	56.7	55.8	1.961	0.375
Global CBF^1^	32.66 ± 7.35	31.94 ± 10.17	0.257	0.612
Total GM CBF^1^	72.98 ± 21.69	74.23 ± 23.20	0.184	0.669
Cortical CBF^1^	81.83 ± 21.95	82.28 ± 24.40	0.344	0.710
Subcortical GM CBF^1^	72.63 ± 20.81	75.03 ± 23.44	0.481	0.619
ACA GM CBF^1,2^	71.95 ± 17.89	72.05 ± 21.68	0.285	0.753
MCA GM CBF^1,2^	82.36 ± 24.14	84.16 ± 25.56	0.412	0.663
PCA GM CBF^1,2^	87.44 ± 22.91	86.23 ± 28.33	0.289	0.749

*CBF, cerebral blood flow; GM, gray matter; ACA, anterior cerebral artery; MCA, middle cerebral artery; PCA, posterior cerebral artery; WM, white matter; MZ, monozygotic; DZ, dizygotic. Notes: ^1^All CBF estimates are in the unit of mL/100 g/min. ^2^GM CBF in ACA, MCA and PCA includes both cortical and subcortical regions in the corresponding arterial territories*.

### MRI Acquisition

Since this was a multicentric study, three scanners were used for data acquisition. Importantly, a twin pair was always scanned on the same scanner.

#### New South Wales Site

PCASL scans were acquired using a Philips 3T Achieva Quasar Dual scanner (Philips Medical Systems, Netherlands). The acquisition parameters were TR/TE = 4,500/12 ms, label duration = 1,800 ms, post label delay = 2,000 ms, flip angle = 90°, imaging matrix = 128 × 128, and FOV = 240 × 240 × 95 mm^3^. Sixteen slices with slice thickness of 5 mm and 1 mm gap between adjacent slices were acquired. Thirty control-tag pairs (i.e., 60 volumes) were scanned, with background suppression enabled. A separate M0 image without background suppression was also acquired with TR/TE = 6,000/12 ms and the same spatial resolution as the 30 control-tag pairs. T1-weighted scans were also acquired for the postprocessing. The scanning parameters were TR/TE = 6.5/3.0 ms, flip angle = 8°, FOV = 250 × 250 × 190 mm^3^, spatial resolution = 1 mm isotrophic, and matrix size = 256 × 256.

#### Victoria and Queensland Sites

Both Victoria and Queensland study centers have used the same scanner model and identical scanning parameters for ASL and T1. At both sites, PASL scans were acquired from 3T Siemens Magnetom Trio scanners, using the PICORE Q2T perfusion mode. The acquisition parameters were TR/TE = 2,500/11 ms, TI1/TI2 = 700/1,800 ms, flip angle = 90°, phase partial Fourier factor = 7/8, bandwidth = 2232 Hz/pix, imaging matrix = 64 × 64, and FOV = 192 mm. Eleven sequential 6-mm thick slices with a distance factor (i.e., gap) of 25% between adjacent slices were acquired for each volume. The first of the 101 PASL volumes was used as the M0 image. T1-weighted images were acquired in Victoria and Queensland sites with TR/TE/TI = 2,300/2.98/900 ms, flip angle = 9°, 208 sagittal slices, within plane FOV = 256 × 240 mm^2^, voxel size = 1 × 1 × 1 mm^3^, and bandwidth = 240 Hz/pix.

### ASL Image Processing

We applied oxford_asl (Chappell et al., 2009) from the FSL group to process the ASL data and generate CBF maps. Briefly, the latest version of oxford_asl (Release 3.9.13) was used to process ASL data on a CentOS 7 workstation. We applied motion correction with mcflirt, adaptive spatial smoothing (Groves et al., 2009), and partial volume correction (Chappell et al., 2011). CBF measurement with voxel-by-voxel calibration, as recommended by the white paper (Alsop et al., 2015), was reported in the main text. Ventricular CSF and white matter (WM) calibrations were also conducted to validate the findings. Six example perfusion-weighted images (three from PCASL and three from PASL) were shown in Supplementary Figure S1.

After preprocessing and obtaining the CBF maps, both cortical and subcortical GM masks as well as the arterial territory atlas in MNI space (Supplementary Figures S2–S4) were warped to individual T1 space, and then linearly registered to individual ASL space to extract the regional CBF. We calculated regional CBF in individual space instead of MNI space to minimize the ASL signal loss due to non-linear warping and interpolation. The cortical and subcortical GM masks were extracted from the Harvard-Oxford cortical and subcortical structural atlas (Frazier et al., 2005). Due to the relatively low SNR of ASL data and the partial volume issues, we merged all subcortical GM regions, including bilateral thalami, caudate, putamen, pallidum, hippocampi, amygdala and accumbens, to form the subcortical mask. Similarly, the cortical mask was established by combining all cortical regions. The arterial territory masks were built from the atlas presented in Wen and Sachdev (2004), by extracting and merging regions belonging to each of the three main cerebral arteries (ACA, MCA, PCA).

Since WM has lower amount of blood flow and prolonged arterial transit time, CBF estimate in WM regions is of generally lower SNR compared to that in GM on ASL scans (Alsop et al., 2015). Moreover, the significant partial volume problem may also overwhelm ASL signal within WM, and make WM CBF from ASL uninterpretable. Due to the difficulty in accurately quantifying perfusion in WM, we only reported results for GM regions in the current study (except that we included global CBF for the interest of readers). The GM mask was created by linearly registering the GM segmentation (from SPM12) from individual T1 space to ASL space. Regional GM CBF was calculated by further applying the cortical/subcortical and arterial territory masks described previously.

### Statistical Analyses

Univariate analysis of variance (ANOVA) were conducted to test the differences in age and CBF estimates between MZ and DZ. Cross-tabulation with chi-square analysis was carried out to test the differences in gender distribution between MZ and DZ. General linear model with repeated measures (*post hoc* test enabled) was used to examine the differences in GM CBF between ACA, MCA and PCA, as well as between cortical and subcortical GM regions.

CBF residuals after adjusting for age, sex and scanner were calculated using linear regression to harmonize data from different study sites and different ASL types (Supplementary Figure S5). Structural equation modeling (SEM) was then applied to study the heritability and GCs of the adjusted CBF using OpenMx 2.3.1 (Neale et al., 2016). To estimate the heritability of global and regional CBF, the univariate Cholesky ACE model (A, additive genetic component; C, shared environmental component; E, unique environmental component) was fitted, and compared with the AE sub-model for the test of model parsimony. The heritability was also calculated in PCASL and PASL sub-samples separately to examine any differences between the two ASL types. Genetic and environmental correlations (ECs) among the GM CBF in ACA, MCA and PCA territories were computed using multivariate SEM. Similarly, we fitted the full Cholesky ACE model, and for model parsimony, we further tested the Cholesky AE model. Individual and common pathways were also examined for the comparison with Cholesky models.

## Results

### Demographic Characteristics and CBF Estimates

**Figure 1:**
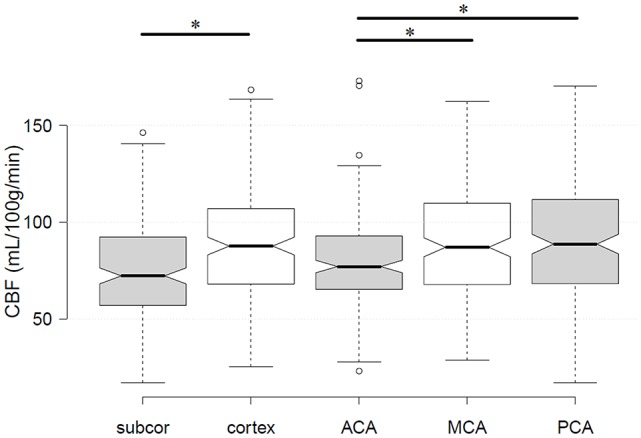
Differences in gray matter (GM) cerebral blood flow (CBF) between subcortical and cortical regions, as well as among the three cerebral arteries. The star label* showed statistically significant differences. Subcor, subcortical GM regions; ACA, anterior cerebral artery; MCA, middle cerebral artery; PCA, posterior cerebral artery.

The demographic characteristics and CBF estimates were summarized in Table 1. The participants of the current study had an age range of 65–93 years (MZ, 65–88 years; DZ, 65–93 years). None of the demographic or CBF measures was significantly different between MZ and DZ (all *p* > 0.05). Cortex had higher CBF than subcortical GM regions (*p* < 0.001; Figure 1). GM CBF was significantly lower in the ACA territory than that in MCA (*p* < 0.001) and PCA (*p* < 0.001; Figure 1). The differences in GM CBF between MCA and PCA were not statistically significant (*p* = 0.403).

### Heritability of CBF

Heritability of global and regional CBF was summarized in Table 2, where all the results were obtained from the most parsimonious AE model. We found a moderate heritability of global CBF (*h*^2^ = 0.611, 95% CI = 0.380–0.761, *p* = 2.19 × 10^−5^). The heritability of examined regions of interest (ROIs) ranged from 0.500 (subcortical GM) to 0.612 (ACA GM). Separate heritability estimates in PCASL and PASL sub-samples were summarized in Supplementary Table S2. As hypothesized, PASL showed lower heritability compared to PCASL, primarily due to greater noise and therefore lower SNR in PASL data.

**Table 2:** Heritability of CBF.

	H2 (95% CI)	$P_{\text{h2}}^{\text{†}}$	ICC MZ (95% CI)	ICC DZ (95% CI)	$P_{\text{AE}}^{‡}$
Whole brain	0.611 (0.380–0.761)	2.19E-5	0.611 (0.380–0.761)	0.306 (0.190–0.380)	0.329
Total GM	0.578 (0.330–0.741)	8.46E-5	0.578 (0.330–0.741)	0.289 (0.165–0.371)	0.650
Cortex	0.593 (0.349–0.751)	5.87E-5	0.593 (0.349–0.751)	0.296 (0.175–0.375)	0.475
Subcortical GM	0.500 (0.182–0.707)	3.68E-3	0.500 (0.182–0.707)	0.250 (0.091–0.354)	1
ACA GM	0.612 (0.378–0.762)	2.52E-5	0.612 (0.378–0.762)	0.306 (0.189–0.381)	0.405
MCA GM	0.595 (0.349–0.754)	5.83E-5	0.595 (0.349–0.754)	0.298 (0.175–0.377)	0.754
PCA GM	0.554 (0.289–0.728)	3.09E-4	0.554 (0.289–0.728)	0.277 (0.145–0.364)	0.474

*^†^P-values for the significance of heritability estimates are calculated by comparing AE and E models. ^‡^P-values for the likelihood ratio test comparing ACE with AE model. CBF, cerebral blood flow; GM, gray matter; ACA, anterior cerebral artery; MCA, middle cerebral artery; PCA, posterior cerebral artery; WM, white matter; H2, heritability; CI, confidence interval; ICC, intra-class correlation*.

Pearson correlations of the CBF measures derived from three different calibration tissues (voxel-by-voxel, WM, ventricular CSF) have been shown in Supplementary Table S3. The CBF measures were highly correlated among the three calibrations. Heritability of CBF calibrated with WM and ventricular CSF has been summarized in Supplementary Table S4. The results largely replicated the findings using voxel-by-voxel calibration. The age moderation on CBF heritability estimates was shown in Supplementary Figures S6, S7 (see Supplementary Text for detailed results).

### Genetic Correlations of Regional CBF

**Figure 2:**
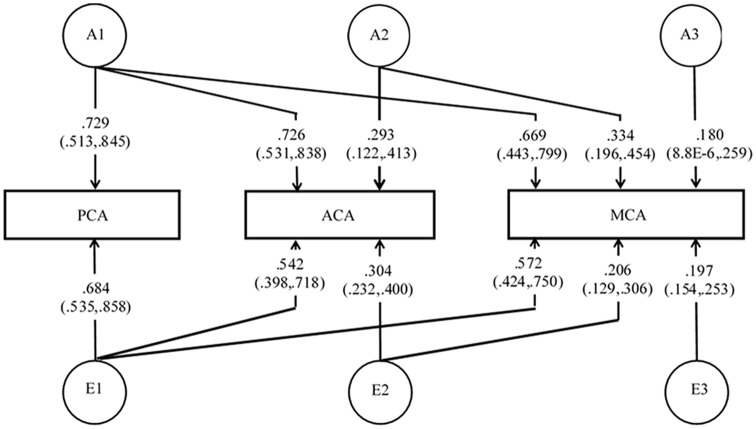
Multivariate Cholesky model for genetic and environmental relationships between PCA, ACA, and MCA. Since PCA has a different origin (basilar artery) from ACA and MCA (internal carotid artery), we also tested whether there were divergent genetic and environmental contributions to the anterior (ACA and MCA) and posterior (PCA) circulations.

The multivariate model fitting has been summarized in Supplementary Table S5. Since ACA and MCA were originated from internal carotid artery, whereas PCA was an extension of the basilar artery, we arrange the three arteries in the order of PCA, ACA, MCA in the Cholesky ACE model. The Cholesky AE model provided a comparable fit to the full Cholesky ACE model. For model parsimony, Cholesky AE model was used to examine GCs, due to the lower number of parameters (15 in Cholesky AE model vs. 21 in Cholesky ACE model), lower Akaike information criterion (−195.86 in Cholesky AE model vs. −188.56 in Cholesky ACE model), and high *p*-value (0.582) in model comparison. In Cholesky AE model, the heritability estimates (additive genetic variance) of PCA, ACA and MCA were 0.532 (95% CI, 0.263–0.714), 0.614 (95% CI, 0.381–0.763) and 0.592 (95% CI, 0.347–0.752), which were in line with the estimates from the univariate AE model. The path coefficients for the Cholesky AE model have been shown in Figure 2. The A1 factor provided similar additive genetic contribution to all three cerebral arteries. Similarly, A2 had comparable genetic influences on ACA and MCA. The effects of the third genetic component (A3) on MCA was not significant. GC analyses using the Cholesky AE model showed that the GM CBF in the three arterial territories (ACA, MCA, PCA) shared similar genetic and environmental effects (GC = 0.870–0.969; EC = 0.872–0.938; Table 3). The common pathway AE model also showed a similar fit as the Cholesky AE model (Supplementary Table S5), and its path coefficients were shown in Supplementary Figure S8. The heritability estimates and GC using the common pathway AE model were comparable to the Cholesky AE model. The strong contributions from the latent factor supported the convergent genetic and environmental effects found in Cholesky AE model.

**Table 3:** Genetic correlations of CBF between cortical and subcortical GM, and between the GM regions of cerebral arterial territories.

	Genetic correlation (95% CI)	Environmental correlation (95% CI)	Phenotype correlation (95% CI)
Cortical and subcortical	0.940 (0.920–0.975)	0.914 (0.850–0.952)	0.929 (0.898–0.951)
ACA and MCA	0.969 (0.943–0.990)	0.938 (0.890–0.966)	0.957 (0.938–0.970)
ACA and PCA	0.927 (0.884–0.988)	0.872 (0.769–0.932)	0.901 (0.858–0.931)
MCA and PCA	0.870 (0.798–0.941)	0.895 (0.808–0.944)	0.879 (0.827–0.916)

*GM, gray matter; ACA, anterior cerebral artery; MCA, middle cerebral artery; PCA, posterior cerebral artery; CI, confidence interval*.

Using bivariate AE model, high genetic and ECs of CBF were also observed between cortical and subcortical GM regions (GC = 0.940; EC = 0.914; Table 3).

## Discussion

CBF has been associated with many aging-related neuropathology, including Alzheimer’s disease (AD; Wang et al., 2013), frontotemporal dementia (Du et al., 2006), and cerebral small vessel disease (Shi et al., 2016). More recent studies have shown the potential of using CBF as a preclinical marker for AD (Wierenga et al., 2014). Being an important biomarker and a potential predictor for aging-related neuropathology, CBF has not been well studied for its genetic basis. An essential step is to define the level of genetic control on CBF, especially in aging population. The current study tried to fulfill this gap. In a cohort of 41 MZ and 25 DZ twin pairs, we studied the genetic contribution to CBF. THe results showed that cortex had higher CBF than subcortical GM structures. CBF in the ACA territory was lower than MCA and PCA territories. Global CBF was moderately heritable in older adults. Heritability did not vary much across the examined cerebral ROIs. Cortical and subcortical GM CBF shared largely common genetic effects. The GCs of GM CBF among the three arterial territories (ACA, MCA, PCA) were also high. In conclusion, the findings suggested a convergent genetic basis of the CBF in cerebral GM in older adults. Taken together with the close relationship between CBF and aging-related neuropathology, the findings warranted future studies to examine whether the genetic factors regulating cerebral perfusion in aging contributes to aging-related neuropathology.

The anterior circulation (ACA and MCA) originating from internal carotid artery, accounts for approximately 72% of total CBF (Zarrinkoob et al., 2015). The CBF in the ACA territory is approximately half of that in MCA. The current study found lower CBF in ACA, compared with MCA and PCA. The current finding in community-based aging population is in line with previous studies showing lower CBF velocity in ACA, but not in other major arteries (Beishon et al., 2017). Our finding is also comparable to the CBF distribution shown in a previous study (Zarrinkoob et al., 2015), which included both young and elderly participants, except for a slightly higher CBF in the posterior circulation in our results.

In the current study, cortex was found to have higher CBF than subcortical GM regions. Subcortical areas are irrigated by penetrating branches originating from the arteries near the Circle of Willis at the base of the brain. As a result, it is expected that blood supply in subcortical GM is lower than the cortex. However, even though we applied partial volume correction to minimize the effects, we cannot exclude the possible contributions from partial volume artifacts to the quantification of subcortical GM CBF, given that subcortical GM structures are of generally smaller size. The slightly lower heritability estimates in subcortical GM than other examined ROIs, may also support this view as the noise in the CBF measurements due to partial volume can decrease the heritability estimates.

In a FAIR imaging study in middle-aged and elderly participants (age range, 41–89 years; ~75% with T2D), Raffield et al. (2015) found the heritability of global GM CBF to be 0.55 ± 0.14 without controlling for any covariates, 0.35 ± 0.14 after adjusting for age and sex, and 0.31 ± 0.14 after further controlling for T2D status. In another study using phase-contrast MRI (age range, 45–97 years; Ikram et al., 2018), only the CBF rate in the whole brain (*h*^2^ (SE) = 0.18 (0.10), *p* = 0.033) and the basilar artery (*h*^2^ (SE) = 0.24 (0.10), *p* = 0.0056) were significantly heritable. Our heritability estimates are slightly higher than these two prior studies. The increased heritability of CBF with age (Ikram et al., 2018) may partially explain the higher heritability estimation in the current study, given that the participants included the current study were all older adults (age range, 65–93 years; Table 1). The current finding of the decreasing heritability estimates of global CBF (Supplementary Figures S6, S7; Supplementary Text) may suggest a non-linear change in CBF heritability from middle to old age, which needs future study to confirm. Furthermore, cerebral blood perfusion (mL/100 g/min) measures the amount of blood perfusing into the brain tissue within a unit of time, which is to some extent different from CBF rate (mL/s) within blood vessel used in Ikram et al. (2018). The moderate heritability of global CBF in the current findings is comparable to previous results regarding the heritability of myocardial blood flow (Su et al., 2012) and blood pressure (Hottenga et al., 2005; Kupper et al., 2005).

The current study showed strong GCs in CBF between cortical and subcortical GM regions, as well as among the three arterial territories, indicating a convergent genetic control for the blood perfusion in cerebral GM. This was supported by the strong effects from the latent factor in the common pathway model (Supplementary Figure S8). This is in line with previous findings on the uniform genetic effects on brain function-related phenotypes (Heck et al., 2014; Johnson et al., 2016).

The fundamental differences in the labeling techniques between PCASL and PASL, and the consequent differences in the SNR and image quality, might have impact on the current findings, as indicated by the varied heritability estimates between PCASL and PASL (Supplementary Table S2). Macrovascular artifacts may be different in the data generated by the two labeling methods, and with single TI ASL data, the macrovascular contamination may not be properly corrected. Moreover, the variation in bolus arrival time across different brain regions is not able to be adjusted for in single TI ASL data, especially PASL. Adding PASL data to PCASL data seemed to underestimate heritability estimates as shown in Table 2 and Supplementary Table S2. The ASL white paper has suggested lower SNR in PASL data (Alsop et al., 2015), which was observed as the higher variance in the PASL CBF measures (Supplementary Table S2). Since heritability estimates depend on the variance of the phenotype, this may at least partially explain the underestimated CBF heritability in the combined sample. In the current study, we have made our best efforts to account for the variation in ASL types and the CBF measures; residuals were calculated to minimize the effects of scanner and demographic factors (age and sex; Supplementary Figure S5). In addition, each twin pair was scanned on the same scanner. Therefore, the differences were more likely to be between-twin-pair rather than within-twin-pair, and heritability estimates would depend on within-twin-pair co-variances. The relatively small sample size included in the current study may also cause a relatively large confidence interval (CI) in the heritability estimates. Studies with larger sample sizes are necessary to confirm the current findings. Despite the limitations, the current work provided new evidence, with a well-controlled twin cohort, to the research of genetic basis of CBF in the aging brain, and warranted further work to uncover the genetic determinants for CBF.

## Data Availability

All datasets generated for this study are included in the manuscript and/or the Supplementary Files.

## Ethics Statement

OATS has been approved by the ethics committees of the Australian Twin Registry, University of New South Wales, University of Melbourne, Queensland Institute of Medical Research, and the South Eastern Sydney & Illawarra Area Health Service. Informed consent was obtained from all participants, and the methods were carried out in accordance with the relevant guidelines.

## Author Contributions

JJ, WW, AT and PS designed the study and interpreted the data. JJ, FK and TL processed the ASL data. JJ drafted the manuscript. FK, WW, PS and QX revised the manuscript critically for important intellectual content. PS, JT, DA, MW and VC acquired the data. All authors read, edited and approved the manuscript.

## Conflict of Interest Statement

The authors declare that the research was conducted in the absence of any commercial or financial relationships that could be construed as a potential conflict of interest.

## References

[B1] Alsop D. C. Detre J. A. Golay X. Gunther M. Hendrikse J. Hernandez-Garcia L.. (2015). Recommended implementation of arterial spin-labeled perfusion MRI for clinical applications: a consensus of the ISMRM perfusion study group and the European consortium for ASL in dementia. Magn. Reson. Med. 73, 102–116. 10.1002/mrm.25197 24715426 PMC4190138

[B2] Attwell D. Laughlin S. B. (2001). An energy budget for signaling in the grey matter of the brain. J. Cereb. Blood Flow Metab. 21, 1133–1145. 10.1097/00004647-200110000-00001 11598490

[B3] Beishon L. Haunton V. J. Panerai R. B. Robinson T. G. (2017). Cerebral hemodynamics in mild cognitive impairment: a systematic review. J. Alzheimers Dis. 59, 369–385. 10.3233/jad-170181 28671118

[B4] Biagi L. Abbruzzese A. Bianchi M. C. Alsop D. C. Del Guerra A. Tosetti M. (2007). Age dependence of cerebral perfusion assessed by magnetic resonance continuous arterial spin labeling. J. Magn. Reson. Imaging 25, 696–702. 10.1002/jmri.20839 17279531

[B5] Borogovac A. Asllani I. (2012). Arterial spin labeling (ASL) fMRI: advantages, theoretical constrains, and experimental challenges in neurosciences. Int. J. Biomed. Imaging 2012:818456. 10.1155/2012/818456 22966219 PMC3432878

[B6] Chappell M. A. Groves A. R. MacIntosh B. J. Donahue M. J. Jezzard P. Woolrich M. W. (2011). Partial volume correction of multiple inversion time arterial spin labeling MRI data. Magn. Reson. Med. 65, 1173–1183. 10.1002/mrm.22641 21337417

[B7] Chappell M. A. Groves A. R. Whitcher B. Woolrich M. W. (2009). Variational Bayesian inference for a nonlinear forward model. IEEE Trans. Sig. Proc. 57, 223–236. 10.1109/tsp.2008.2005752

[B8] Chen J. J. Rosas H. D. Salat D. H. (2011). Age-associated reductions in cerebral blood flow are independent from regional atrophy. Neuroimage 55, 468–478. 10.1016/j.neuroimage.2010.12.032 21167947 PMC3435846

[B9] Cipolla M. J. (2009). The Cerebral Circulation. San Rafael, CA: Morgan and Claypool Life Sciences. 21452434

[B10] Coutinho J. M. Derkatch S. Potvin A. R. Tomlinson G. Casaubon L. K. Silver F. L.. (2017). Carotid artery web and ischemic stroke: a case-control study. Neurology 88, 65–69. 10.1212/WNL.0000000000003464 27864523 PMC5200857

[B11] Dastur D. K. (1985). Cerebral blood-flow and metabolism in normal human aging, pathological aging, and senile dementia. J. Cereb. Blood Flow Metab. 5, 1–9. 10.1038/jcbfm.1985.1 3972914

[B12] Du A. T. Jahng G. H. Hayasaka S. Kramer J. H. Rosen H. J. Gorno-Tempini M. L.. (2006). Hypoperfusion in frontotemporal dementia and Alzheimer disease by arterial spin labeling MRI. Neurology 67, 1215–1220. 10.1212/01.wnl.0000238163.71349.78 17030755 PMC1779761

[B13] Farkas E. Luiten P. G. (2001). Cerebral microvascular pathology in aging and Alzheimer’s disease. Prog. Neurobiol. 64, 575–611. 10.1016/s0301-0082(00)00068-x 11311463

[B14] Frazier J. A. Chiu S. Breeze J. L. Makris N. Lange N. Kennedy D. N.. (2005). Structural brain magnetic resonance imaging of limbic and thalamic volumes in pediatric bipolar disorder. Am. J. Psychiatry 162, 1256–1265. 10.1176/appi.ajp.162.7.1256 15994707

[B15] Groves A. R. Chappell M. A. Woolrich M. W. (2009). Combined spatial and non-spatial prior for inference on MRI time-series. Neuroimage 45, 795–809. 10.1016/j.neuroimage.2008.12.027 19162204

[B16] Heck A. Fastenrath M. Ackermann S. Auschra B. Bickel H. Coynel D.. (2014). Converging genetic and functional brain imaging evidence links neuronal excitability to working memory, psychiatric disease, and brain activity. Neuron 81, 1203–1213. 10.1016/j.neuron.2014.01.010 24529980 PMC4205276

[B17] Hottenga J. J. Boomsma D. I. Kupper N. Posthuma D. Snieder H. Willemsen G.. (2005). Heritability and stability of resting blood pressure. Twin Res. Hum. Genet. 8, 499–508. 10.1375/183242705774310123 16212839

[B18] Ighodaro E. T. Abner E. L. Fardo D. W. Lin A. L. Katsumata Y. Schmitt F. A.. (2017). Risk factors and global cognitive status related to brain arteriolosclerosis in elderly individuals. J. Cereb. Blood Flow Metab. 37, 201–216. 10.1177/0271678x15621574 26738751 PMC5363738

[B19] Ikram M. A. Zonneveld H. I. Roshchupkin G. Smith A. V. Franco O. H. Sigurdsson S.. (2018). Heritability and genome-wide associations studies of cerebral blood flow in the general population. J. Cereb. Blood Flow Metab. 38, 1598–1608. 10.1177/0271678X17715861 28627999 PMC6120124

[B20] Johnson M. R. Shkura K. Langley S. R. Delahaye-Duriez A. Srivastava P. Hill W. D.. (2016). Systems genetics identifies a convergent gene network for cognition and neurodevelopmental disease. Nat. Neurosci. 19, 223–232. 10.1038/nn.4205 26691832

[B21] Kupper N. Willemsen G. Riese H. Posthuma D. Boomsma D. I. de Geus E. J. C. (2005). Heritability of daytime ambulatory blood pressure in an extended twin design. Hypertension 45, 80–85. 10.1161/01.hyp.0000149952.84391.54 15557390

[B22] Lee C. Lopez O. L. Becker J. T. Raji C. Dai W. Y. Kuller L. H.. (2009). Imaging cerebral blood flow in the cognitively normal aging brain with arterial spin labeling: implications for imaging of neurodegenerative disease. J. Neuroimaging 19, 344–352. 10.1111/j.1552-6569.2008.00277.x 19292827 PMC2755631

[B23] Lu H. Xu F. Rodrigue K. M. Kennedy K. M. Cheng Y. Flicker B.. (2011). Alterations in cerebral metabolic rate and blood supply across the adult lifespan. Cereb. Cortex 21, 1426–1434. 10.1093/cercor/bhq224 21051551 PMC3097991

[B24] Marchal G. Rioux P. Petit-Taboue M. C. Sette G. Travere J. M. Le Poec C.. (1992). Regional cerebral oxygen consumption, blood flow, and blood volume in healthy human aging. Arch. Neurol. 49, 1013–1020. 10.1001/archneur.1992.00530340029014 1417508

[B25] Neale M. C. Hunter M. D. Pritikin J. N. Zahery M. Brick T. R. Kirkpatrick R. M.. (2016). OpenMx 2.0: extended structural equation and statistical modeling. Psychometrika 81, 535–549. 10.1007/s11336-014-9435-8 25622929 PMC4516707

[B26] Raffield L. M. Cox A. J. Hugenschmidt C. E. Freedman B. I. Langefeld C. D. Williamson J. D.. (2015). Heritability and genetic association analysis of neuroimaging measures in the diabetes heart study. Neurobiol. Aging 36, 1602.e7–1615.e7. 10.1016/j.neurobiolaging.2014.11.008 25523635 PMC4346514

[B27] Sachdev P. S. Lammel A. Trollor J. N. Lee T. Wright M. J. Ames D.. (2009). A comprehensive neuropsychiatric study of elderly twins: the Older Australian twins study. Twin Res. Hum. Genet. 12, 573–582. 10.1375/twin.12.6.573 19943720

[B28] Shi Y. Thrippleton M. J. Makin S. D. Marshall I. Geerlings M. I. de Craen A. J. M.. (2016). Cerebral blood flow in small vessel disease: a systematic review and meta-analysis. J. Cereb. Blood Flow Metab. 36, 1653–1667. 10.1177/0271678x16662891 27496552 PMC5076792

[B29] Su S. Votaw J. Faber T. Khan D. Bremner J. D. Goldberg J.. (2012). Measurement of heritability of myocardial blood flow by positron emission tomography: the twins heart study. Heart 98, 495–499. 10.1136/heartjnl-2011-301080 22323242 PMC4380432

[B30] Tarumi T. Zhang R. (2018). Cerebral blood flow in normal aging adults: cardiovascular determinants, clinical implications and aerobic fitness. J. Neurochem. 144, 595–608. 10.1111/jnc.14234 28986925 PMC5874160

[B31] Wang Z. Das S. R. Xie S. X. Arnold S. E. Detre J. A. Wolk D. A.. (2013). Arterial spin labeled MRI in prodromal Alzheimer’s disease: a multi-site study. Neuroimage Clin. 2, 630–636. 10.1016/j.nicl.2013.04.014 24179814 PMC3777751

[B32] Wen W. Sachdev P. (2004). The topography of white matter hyperintensities on brain MRI in healthy 60- to 64-year-old individuals. Neuroimage 22, 144–154. 10.1016/j.neuroimage.2003.12.027 15110004

[B33] Wierenga C. E. Clark L. R. Dev S. I. Shin D. D. Jurick S. M. Rissman R. A.. (2013). Interaction of age and APOE genotype on cerebral blood flow at rest. J. Alzheimers Dis. 34, 921–935. 10.3233/jad-121897 23302659 PMC4124882

[B34] Wierenga C. E. Hays C. C. Zlatar Z. Z. (2014). Cerebral blood flow measured by arterial spin labeling MRI as a preclinical marker of Alzheimer’s disease. J. Alzheimers Dis. 42, S411–S419. 10.3233/jad-141467 25159672 PMC5279221

[B35] Xu G. F. Rowley H. A. Wu G. H. Alsop D. C. Shankaranarayanan A. Dowling M. (2010). Reliability and precision of pseudo-continuous arterial spin labeling perfusion MRI on 3.0 T and comparison with ^15^O-water PET in elderly subjects at risk for Alzheimer’s disease. NMR Biomed. 23, 286–293. 10.1002/nbm.1462 19953503 PMC2843795

[B36] Zarrinkoob L. Ambarki K. Wahlin A. Birgander R. Eklund A. Malm J. (2015). Blood flow distribution in cerebral arteries. J. Cereb. Blood Flow Metab. 35, 648–654. 10.1038/jcbfm.2014.241 25564234 PMC4420884

[B37] Zhang N. Gordon M. L. Goldberg T. E. (2017). Cerebral blood flow measured by arterial spin labeling MRI at resting state in normal aging and Alzheimer’s disease. Neurosci. Biobehav. Rev. 72, 168–175. 10.1016/j.neubiorev.2016.11.023 27908711

