# Supplementary material for: Cerebral Blood Flow in Community-Based Older Twins Is Moderately Heritable: An Arterial Spin Labeling Perfusion Imaging Study
Source: Front Aging Neurosci. 2019 Jul 2;11:169. doi: 10.3389/fnagi.2019.00169 (PMC6615405; doi:10.3389/fnagi.2019.00169)
Supplement: Supplementary file 1 [file Data_Sheet_1.docx]

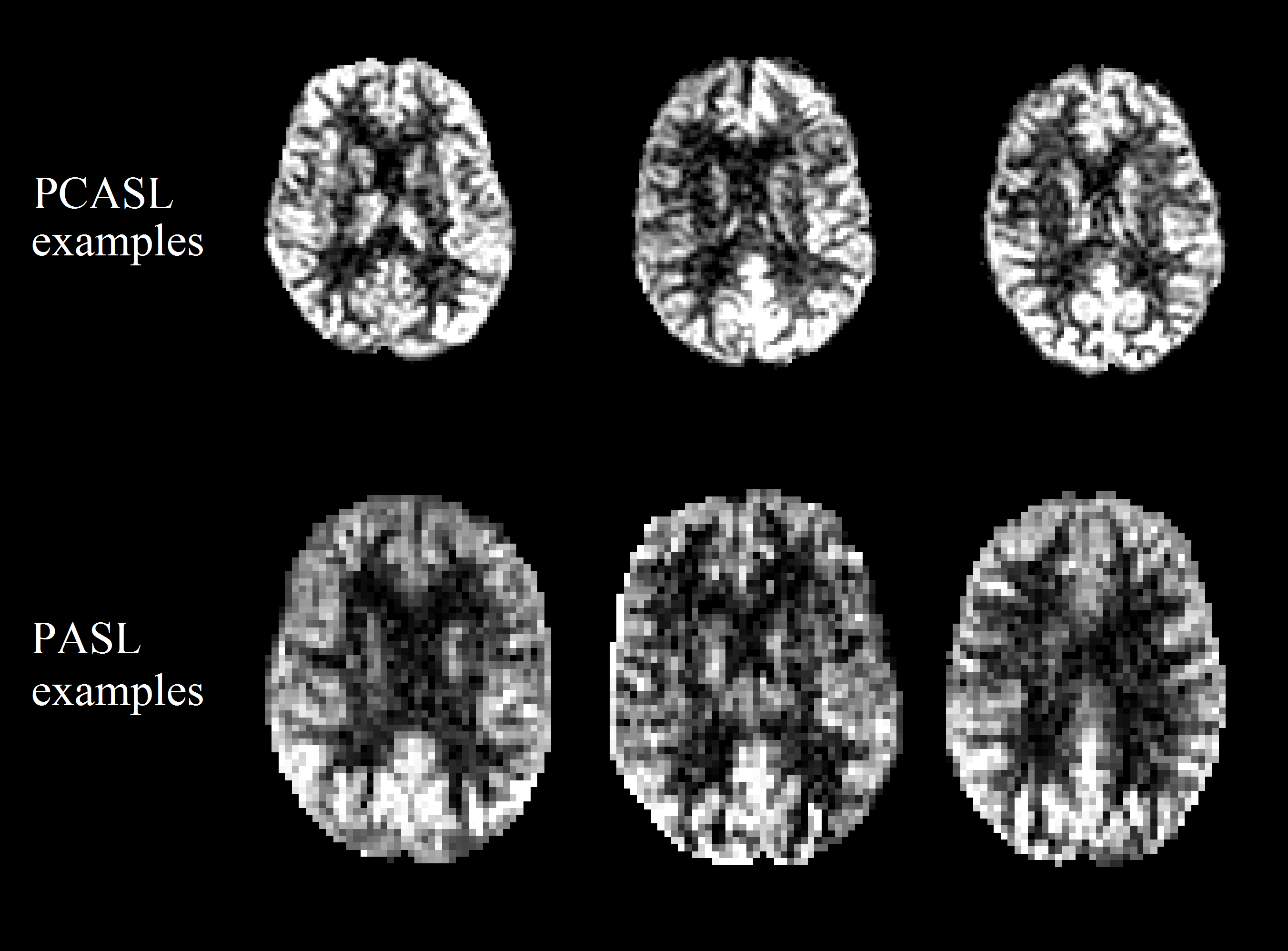


Supplementary Figure 1. Six example perfusion-weighted images derived from three pseudo-continuous ASL (PCASL) and three pulsed ASL (PASL) scans.


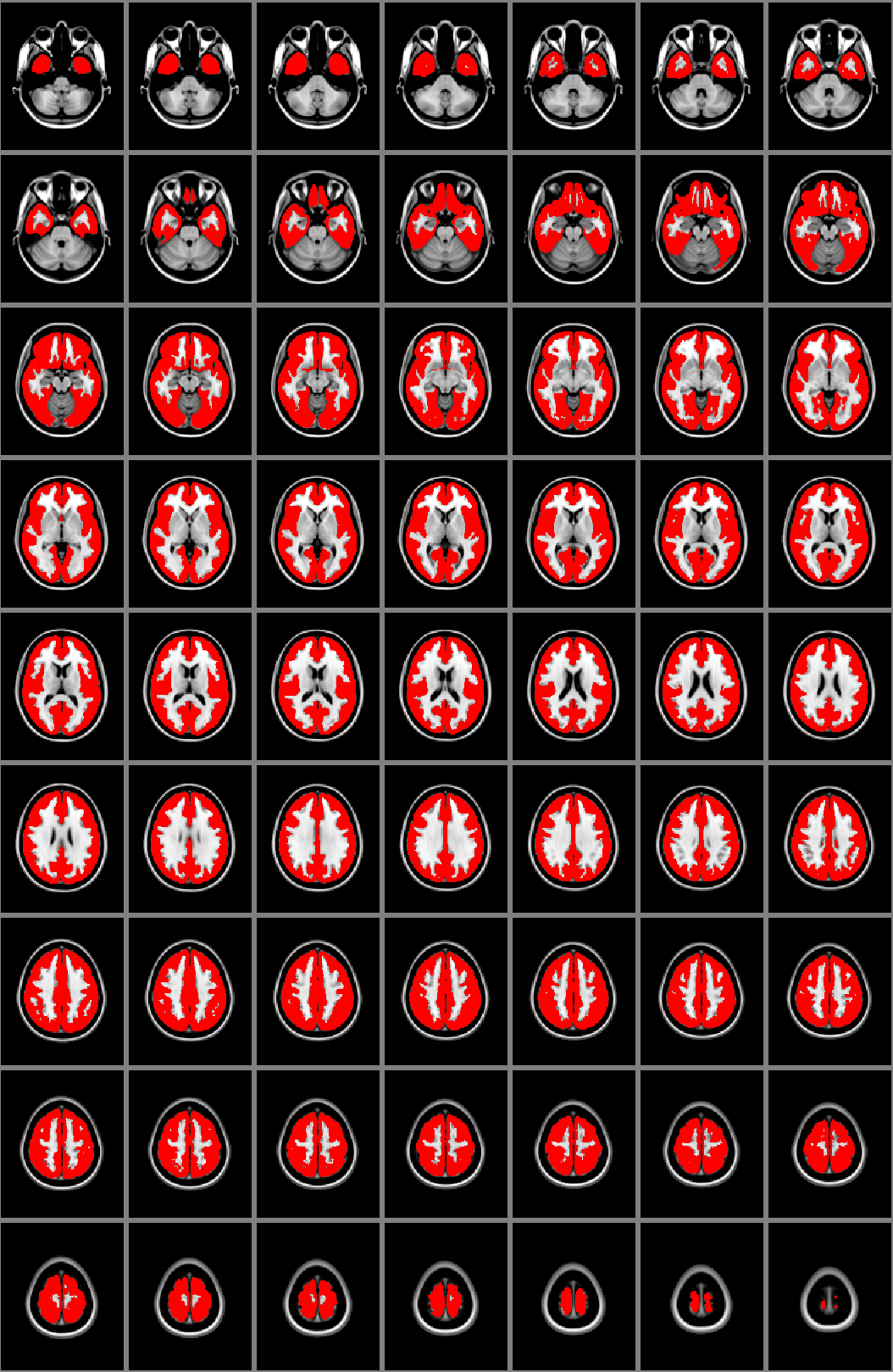


Supplementary Figure 2. Cortical mask in MNI space.


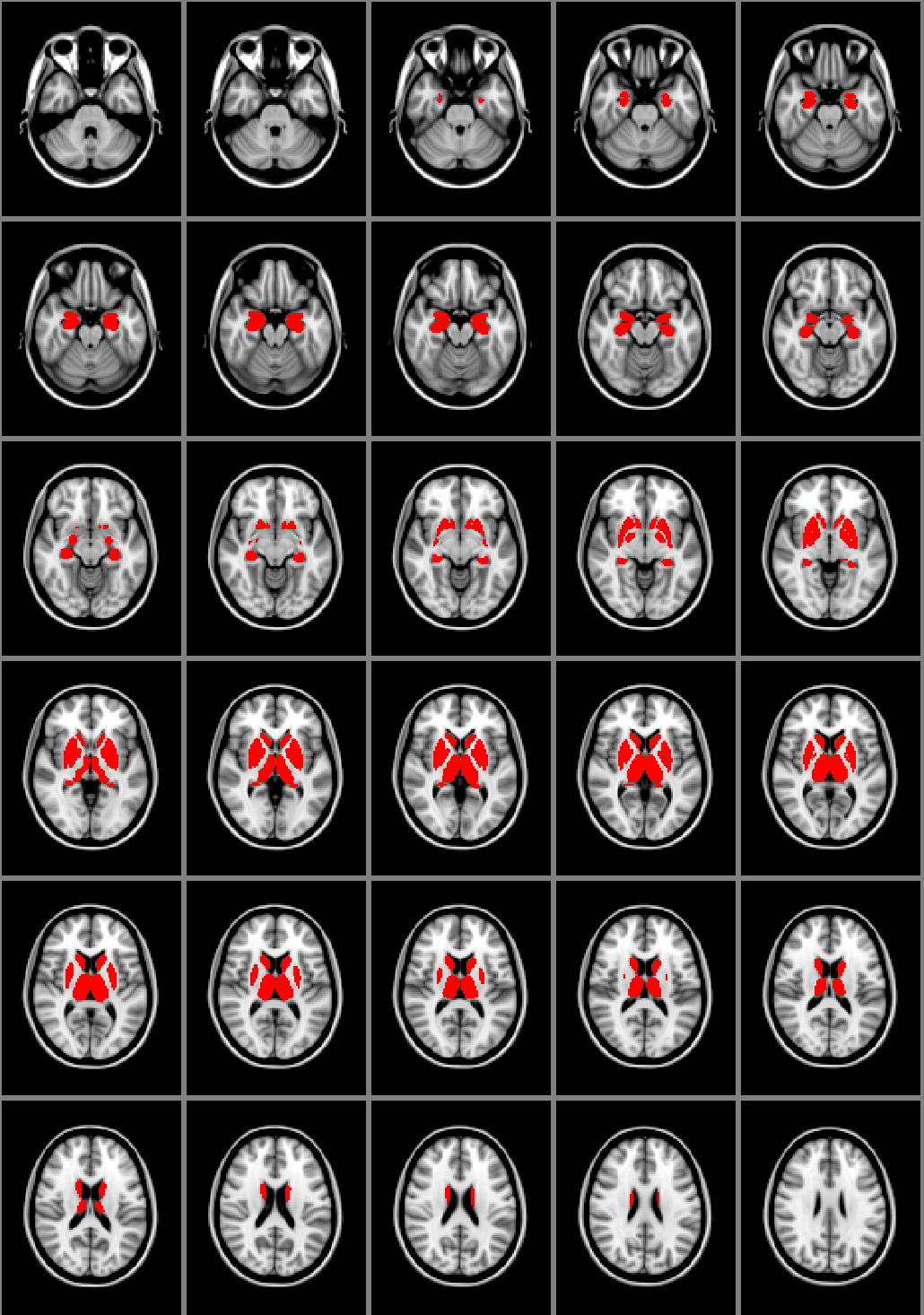


Supplementary Figure 3. Subcortical grey matter mask in MNI space.


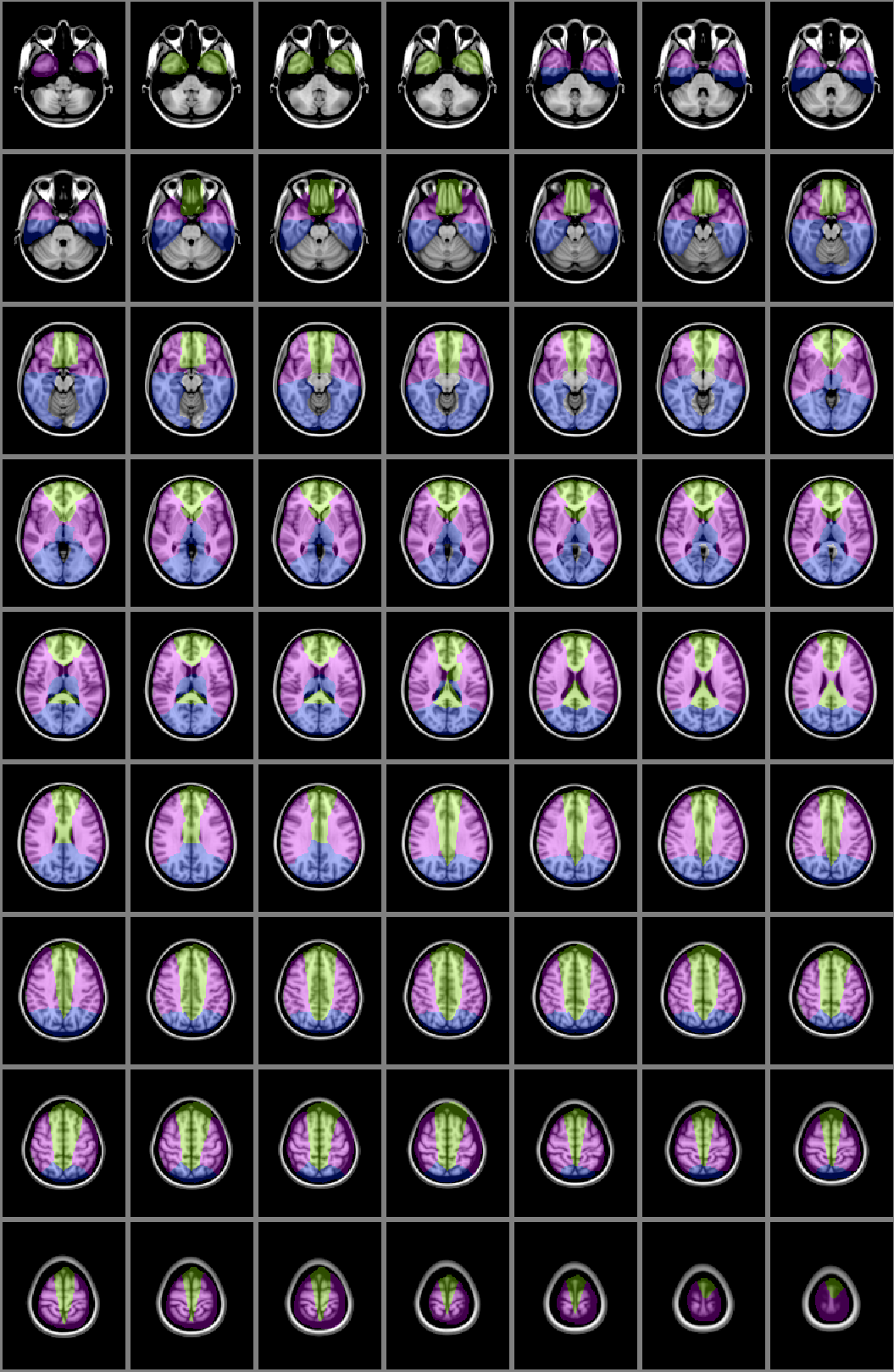


Supplementary Figure 4. Arterial territory atlas in MNI space. Green = anterior cerebral artery; purple = middle cerebral artery; blue = posterior cerebral artery.

(A)

(B)

Supplementary Figure 5. Principal component analyses (PCA) to explore the stratification of CBF measures. (A) the first two principal components of the raw CBF. Separation between the two ASL types (PCASL from NSW study site, and PASL from QLD and VIC) can be observed. (B) after adjusting for age, sex and scanner, CBF from PCASL and PASL “merge” together, indicating a good standardisation of data.


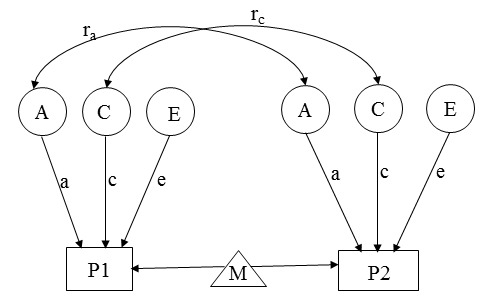


Supplemantary Figure 6. The path diagram for the age moderated ACE twin model. The CBF of the twin 1 (P1) and twin 2 (P2) are modelled as the function of the mean parameter (M) and the latent additive (A), shared environment (C) and environment (E) factors. The mean is further modelled as a function of the *k* covariates $M=\mu+\beta_{1}X_{1}+\ldots+\beta_{k}X_{k}$, where µ is the overall mean of the phenotypes and *X_1,_ X_2,_…,X_k_* are the k covariates ( such as age, sex and scanners) and β_1,_ β2,…, β_k_ are the regression parameters of the model. The path coefficients a, c and e are the estimated loadings of the latent factors, which are further decomposed as a=a_0_+a_1_age; c=c_0_+c_1_age; e=e_0_+e_1_age to accommodate the moderating effects of age. The parameter r_a_ (r_a_=1 for MZ twin pairs and r_a_=0.5 for DZ twin pars) and r_c_ (r_c=1_ for both MZ and DZ twin pars) respectively denote the additive genetic and shared environmental correlation between the twin pairs.

Supplementary Figure 7. Age moderation on CBF heritability estimates (AE model).

A_S2_

E_S2_

A_S3_

E_S3_

A

E

PCA

ACA

MCA

A_S1_

E_S1_

.780

(.604,.874)

.626

(.486,.797)

.952

(.817,1.122)

1.057

(.926,1.225)

1.028

(.896,1.197)

.292

(.162,.399)

9.3E-6

(8.1E-6,.167)

.200

(.115,.266)

.281

(.203,.382)

.164

(.084,.227)

.140

(.040,.218)

Supplementary Figure 8. Common pathway (AE model).

Supplementary Table 1. Cardiovascular conditions of the study sample

|  | **MZ** | **DZ** |
| --- | --- | --- |
| **Framingham cardiovascular disease risk score** | 14.00 ± 3.84 | 14.91 ± 3.59 |
| **Doctor diagnosis of stroke** | 2 / 75  (2.67%) | 1 / 47  (2.13%) |
| **Doctor diagnosis of transient ischaemic attack** | 5 / 74  (6.76%) | 2 / 48  (4.17%) |
| **Doctor diagnosis of arterial myocardial infarction** | 3 / 76  (3.95%) | 1 / 48  (2.08%) |
| **Doctor diagnosis of hypertension** | 35/75  (46.67%) | 23/48  (47.92%) |
| **Taking antihypertensives** | 39/76  (51.32%) | 22/48  (45.83%) |
| **Systolic blood pressure** | 137.90 ± 21.51 | 138.94 ± 17.95 |
| **Diastolic blood pressure** | 81.47 ± 10.68 | 82.43 ± 11.91 |

Supplementary Table 2. Heritability of adjusted CBF using PCASL (NSW study site) and PASL (QLD and VIC study sites)

|  | **PCASL (30 twin pairs)** | | | | | | | **PASL (36 twin pairs)** | | | | | | | |
| --- | --- | --- | --- | --- | --- | --- | --- | --- | --- | --- | --- | --- | --- | --- | --- |
|  | **Whole brain** | **Total GM** | **Cortex** | **Subcortical GM** | **ACA GM** | **MCA GM** | **PCA GM** | **Whole brain** | **Total GM** | **Cortex** | **Subcortical GM** | **ACA GM** | **MCA GM** | **PCA GM** |  |
| **CBF (mL/100g/min)** | 30.20 ± 6.38 | 64.54 ± 12.10 | 75.17 ± 12.89 | 67.36 ± 15.69 | 70.68 ± 13.32 | 69.41 ± 12.16 | 70.78 ± 13.41 | 35.45 ± 10.53 | 87.37 ± 20.79 | 100.87 ± 23.90 | 88.41 ± 21.09 | 86.40 ± 20.84 | 103.06 ± 25.68 | 104.08 ± 26.64 |  |
| **H2 (95% CI)** | 0.758 (0.499-0.881) | 0.758 (0.503-0.881) | 0.763 (0.500-0.885) | 0.727 (0.423-0.868) | 0.777 (0.527-0.892) | 0.713 (0.401-0.861) | 0.789 (0.562-0.896) | 0.337 (5.19E-14-0.629) | 0.397 (0.008-0.679) | 0.381 (0.004-0.659) | 0.325 (2.05E-13-0.649) | 0.334 (4.66E-16-0.620) | 0.487 (0.115-0.733) | 0.242 (3.14E-15-0.571) |  |
| **p-val** | 8.40E-05 | 7.24E-05 | 1.05E-04 | 5.42E-04 | 6.58E-05 | 5.76E-04 | 2.05E-05 | 0.079 | 0.045 | 0.047 | 0.12 | 0.071 | 0.012 | 0.229 |  |
| **MZ_ICC (95% CI)** | 0.758 (0.499-0.881) | 0.758 (0.503-0.881) | 0.763 (0.500-0.885) | 0.727 (0.423-0.868) | 0.777 (0.527-0.892) | 0.713 (0.401-0.861) | 0.789 (0.562-0.896) | 0.337 (5.19E-14-0.629) | 0.397 (0.008-0.679) | 0.381 (0.004-0.659) | 0.325 (2.05E-13-0.649) | 0.334 (4.66E-16-0.620) | 0.487 (0.115-0.733) | 0.242 (3.14E-15-0.571) |  |
| **DZ_ICC (95% CI)** | 0.379 (0.250-0.441) | 0.379 (0.251-0.440) | 0.381 (0.250-0.442) | 0.364 (0.212-0.434) | 0.389 (0.264-0.446) | 0.356 (0.201-0.430) | 0.394 (0.281-0.448) | 0.168 (3.00E-14-0.315) | 0.198 (0.004-0.339) | 0.191 (0.002-0.329) | 0.163 (4.533E-13-0.325) | 0.167 (4.918E-13-0.31) | 0.244 (0.058-0.366) | 0.121 (1.62E-12-0.285) |  |

Supplementary Table 3. Pearson correlations of CBF measures calculated with three different calibration tissues (voxel-by-voxel, WM, and ventricular CSF)

| **Region of interest** | **Calibration tissues** | **Pearson correlation** | **p-value** |
| --- | --- | --- | --- |
| **Whole brain** | **CSF * vox** | 0.843 | <0.001 |
|  | **WM * vox** | 0.866 | <0.001 |
|  | **CSF * WM** | 0.604 | <0.001 |
| **Total GM** | **CSF * vox** | 0.883 | <0.001 |
|  | **WM * vox** | 0.956 | <0.001 |
|  | **CSF * WM** | 0.806 | <0.001 |
| **Cortex** | **CSF * vox** | 0.971 | <0.001 |
|  | **WM * vox** | 0.933 | <0.001 |
|  | **CSF * WM** | 0.836 | <0.001 |
| **Subcortical GM** | **CSF * vox** | 0.975 | <0.001 |
|  | **WM * vox** | 0.949 | <0.001 |
|  | **CSF * WM** | 0.874 | <0.001 |
| **ACA GM** | **CSF * vox** | 0.970 | <0.001 |
|  | **WM * vox** | 0.866 | <0.001 |
|  | **CSF * WM** | 0.754 | <0.001 |
| **MCA GM** | **CSF * vox** | 0.983 | <0.001 |
|  | **WM * vox** | 0.937 | <0.001 |
|  | **CSF * WM** | 0.875 | <0.001 |
| **PCA GM** | **CSF * vox** | 0.944 | <0.001 |
|  | **WM * vox** | 0.954 | <0.001 |
|  | **CSF * WM** | 0.821 | <0.001 |

NOTES: CBF = cerebral blood flow; CSF = calibration with cerebrospinal fluid; vox = voxel-by-voxel calibration; WM = calibration with white matter; GM = grey matter; ACA = anterior cerebral artery; MCA = middle cerebral artery; PCA = posterior cerebral artery.

Supplementary Table 4. Heritability of adjusted CBF ^1^ calibrated with WM and ventricular CSF

| **Calibration tissue** | **Regions of interest** | **H2 (95% CI)** | **P_h2_** | **ICC MZ (95% CI)** | **ICC DZ (95% CI)** | **P_AE_** |
| --- | --- | --- | --- | --- | --- | --- |
| **WM** | **Whole brain** | 0.528 (0.271-0.706) | 2.84E-4 | 0.528 (0.271-0.706) | 0.264 (0.136-0.353) | 0.705 |
|  | **Total GM** | 0.493 (0.221-0.686) | 9.39E-4 | 0.493 (0.221-0.686) | 0.247 (0.110-0.343) | 1 |
|  | **Cortex** | 0.496 (0.219-0.690) | 1.05E-3 | 0.496 (0.219-0.690) | 0.248 (0.110-0.345) | 1 |
|  | **Subcortical GM** | 0.468 (0.145-0.690) | 5.99E-3 | 0.468 (0.145-0.690) | 0.234 (0.073-0.345) | 1 |
|  | **ACA GM** | 0.477 (0.201-0.675) | 1.41E-3 | 0.477 (0.201-0.675) | 0.239 (0.101-0.338) | 1 |
|  | **MCA GM** | 0.560 (0.296-0.735) | 1.96E-4 | 0.560 (0.296-0.735) | 0.280 (0.148-0.367) | 1 |
|  | **PCA GM** | 0.415 (0.110-0.638) | 9.46E-3 | 0.415 (0.110-0.638) | 0.208 (0.055-0.319) | 0.793 |
| **Ventricular CSF** | **Whole brain** | 0.600 (0.365-0.753) | 3.30E-5 | 0.600 (0.365-0.753) | 0.300 (0.183-0.376) | 0.285 |
|  | **Total GM** | 0.593 (0.350-0.751) | 5.20E-5 | 0.593 (0.350-0.751) | 0.296 (0.175-0.375) | 0.584 |
|  | **Cortex** | 0.588 (0.345-0.747) | 5.94E-5 | 0.588 (0.345-0.747) | 0.294 (0.173-0.373) | 0.433 |
|  | **Subcortical GM** | 0.561 (0.282-0.739) | 5.07E-4 | 0.561 (0.282-0.739) | 0.281 (0.141-0.369) | 0.758 |
|  | **ACA GM** | 0.585 (0.342-0.744) | 6.15E-5 | 0.585 (0.342-0.744) | 0.292 (0.171-0.372) | 0.411 |
|  | **MCA GM** | 0.599 (0.357-0.756) | 4.42E-5 | 0.599 (0.357-0.756) | 0.300 (0.178-0.378) | 0.747 |
|  | **PCA GM** | 0.571 (0.321-0.735) | 1.26E-4 | 0.571 (0.321-0.735) | 0.286 (0.161-0.368) | 0.245 |

^1^ adjusted CBF = Residual of CBF after regressing out the effects of age, sex, and scanner.

WM = white matter; CBF = cerebral blood flow; GM = grey matter; H2 = heritability; P_h2_ = p value for the heritability estimates; CI = confidence interval; ICC = intra-class correlation; MZ = monozygotic twins; DZ = dizygotic twins; P_AE_ = p value for the likelihood ratio test between the full ACE model and the parsimonious AE model.

Supplementary Table 5. Model summary for multivariate analyses

| **Model** | **Number of parameters** | **-2LL** | **AIC** | **diffLL** | **diffdf** | **p-value** |
| --- | --- | --- | --- | --- | --- | --- |
| **CholACE** | 21 | 561.44 | -188.56 | - | - | - |
| **CholAE** | 15 | 566.14 | -195.86 | 4.7 | 6 | 0.582 |
| **IndACE** | 21 | 561.46 | -188.54 | 0.02 | 0 | 0 |
| **IndAE** | 15 | 567.21 | -194.79 | 5.77 | 6 | 0.449 |
| **ComACE** | 18 | 567.02 | -190.98 | 5.58 | 4 | 0.233 |
| **ComAE** | 14 | 568.67 | -197.33 | 7.24 | 8 | 0.511 |

Notes: CholACE, full Cholesky ACE model; CholAE, Cholesky AE model; ChoAEsub1, a sub-model removing the path coefficients going from the latent A_1_ and E_1_ factors to ACA from Cholesky AE model; ChoAEsub2, a sub-model removing the path coefficients going from the latent A_1_ and E_1_ factors to MCA from Cholesky AE model; IndACE, independent pathway ACE model; IndAE, independent pathway AE model; ComACE, common pathway ACE model; ComAE, common pathway AE model; -2LL, negative twice log-likelihood; AIC, Akaike information criterion; diffLL, differences in log-likelihood; diffdf, differences in the number of parameters; p-value, comparison with Cholesky ACE model.

**Supplementary Text**

*Heritability of CBF calibrated with CSF and WM*

In the main text of the current report, we used voxel-wise calibrated CBF according to the white paper [1]. Other common calibration methods include calibrating with measures with in WM or CSF regions, which we also implemented (Supplementary Table 4) for interested readers. We first tested the consistency of voxel-wise with WM and CSF calibrated CBF measures by calculating Pearson’s correlation (Supplementary Table 3). Results showed higher correlation (Pearson correlation coefficient = 0.604 – 0.983) in all considered global and regional CBF measures. A simple comparison between CBF measures derived from voxel-wise and WM/CSF calibration showed largely consistent heritability estimates, except for a slightly lower heritability in WM-calibrated results comparing to voxel-wise and CSF calibration. One possibility of this lower WM-calibrated CBF heritability is the lower spatial resolution of the ASL data (i.e. greater partial volume artefacts), and therefore certain GM regions, especially subcortical GM structures, are also included in the WM mask.

*Age moderation on CBF heritability estimates*

The age moderation on CBF heritability estimates has now been shown on Supplementary Figure 7. Generally, more significant age moderation was observed from 75 years of age. Heritability of global CBF showed a largely consistent decrease from 65 to 85 years. The heritability of CBF in all GM regions remained at the level of ~0.5 before a gradual drop starting from 75 years. Age showed opposite effects on the heritability of cortical and subcortical GM CBF, with cortical CBF being more heritable with age, while heritability of subcortical GM CBF inversely correlating with age. After 75 years old, ACA CBF showed a rapid increase in heritability, while heritability of PCA CBF decreased. The heritability of MCA CBF had a gradual increase from 65 to 85 years.

**Supplementary References**

[1] D.C. Alsop, J.A. Detre, X. Golay, M. Gunther, J. Hendrikse, L. Hernandez-Garcia, H. Lu, B.J. MacIntosh, L.M. Parkes, M. Smits, M.J. van Osch, D.J. Wang, E.C. Wong, and G. Zaharchuk, Recommended implementation of arterial spin-labeled perfusion MRI for clinical applications: A consensus of the ISMRM perfusion study group and the European consortium for ASL in dementia. Magn Reson Med 73 (2015) 102-16.
